# Optical trapping of sub-millimeter sized particles and microorganisms

**DOI:** 10.1038/s41598-023-35829-7

**Published:** 2023-05-27

**Authors:** Laurynas Lialys, Justinas Lialys, Alessandro Salandrino, Brian D. Ackley, Shima Fardad

**Affiliations:** 1 Department of Electrical Engineering & Computer Science, University of Kansas, Lawrence, 66045 USA; 2 I2S, Institute for Information Sciences, University of Kansas, Lawrence, 66045 USA; 3 Department of Molecular Biosciences, University of Kansas, Lawrence, 66045 USA

**Keywords:** Applied optics, Optical physics, Optical techniques, Microscopy, Micro-optics

## Abstract

While optical tweezers (OT) are mostly used for confining smaller size particles, the counter-propagating (CP) dual-beam traps have been a versatile method for confining both small and larger size particles including biological specimen. However, CP traps are complex sensitive systems, requiring tedious alignment to achieve perfect symmetry with rather low trapping stiffness values compared to OT. Moreover, due to their relatively weak forces, CP traps are limited in the size of particles they can confine which is about 100 μm. In this paper, a new class of counter-propagating optical tweezers with a broken symmetry is discussed and experimentally demonstrated to trap and manipulate larger than 100 μm particles inside liquid media. Our technique exploits a single Gaussian beam folding back on itself in an asymmetrical fashion forming a CP trap capable of confining small and significantly larger particles (up to 250 μm in diameter) based on optical forces only. Such optical trapping of large-size specimen to the best of our knowledge has not been demonstrated before. The broken symmetry of the trap combined with the retro-reflection of the beam has not only significantly simplified the alignment of the system, but also made it robust to slight misalignments and enhances the trapping stiffness as shown later. Moreover, our proposed trapping method is quite versatile as it allows for trapping and translating of a wide variety of particle sizes and shapes, ranging from one micron up to a few hundred of microns including microorganisms, using very low laser powers and numerical aperture optics. This in turn, permits the integration of a wide range of spectroscopy techniques for imaging and studying the optically trapped specimen. As an example, we will demonstrate how this novel technique enables simultaneous 3D trapping and light-sheet microscopy of *C. elegans* worms with up to 450 µm length.

## Introduction

Lasers allow for unique light-matter interactions leading to strong optical forces, particle manipulation and trapping^1–8^. Optical trapping is a versatile tool with many applications which has allowed for a wealth of fundamental studies, revolutionizing numerous fields of science and engineering since its discovery^9–15^. The most basic yet powerful implementation of optical traps is the single-beam gradient force trap, known as optical tweezers^16–18^. In this method, the trap is formed when a laser beam is focused tight enough such that optical forces exerted on the particle of interest confine it. These forces are in general classified into two main contributions. One is the gradient forces which pull particles with higher refractive index with respect to the background medium into regions with larger laser intensity. The second is scattering forces which mainly push the particles along the beam propagation direction. The latter forces can counteract particle trapping, leading to an unstable trap, especially for larger particles (above 10 μm). Therefore, finding a practical approach to compensate for the adverse effects of the scattering forces is a crucial step in stable optical trapping. One common solution is the use of high-NA (Numerical Aperture) microscope objectives, to tightly focus the beam so that gradient forces increase to the point of outmatching the scattering forces in the axial direction. Such focusing typically requires microscope objectives with numerical apertures exceeding one (hence immersion type). This results in a short working distance, narrow field of view and extreme local intensities which usually conflict with the needs of practical applications, especially in biology. Another approach which can avoid the aforementioned disadvantages is the use of two moderately focused counter-propagating (CP) identical beams^19–30^. Here each beam balances the forward scattering forces of the other, generating axial stability to form a 3D trap between the foci of the two beams. Such optical trapping inside suspensions has been achieved using high-NA objectives^25,26^, low-NA objectives^19,31^, two fibers^20,32–34^, optical mirror traps^35–41^, optical phase conjugation^42^, holographic counter propagating traps^23,37,40^ and standing waves which are ideal to trap nanoparticles^21–24,35–39^. In these CP trapping configurations, since the trapping occurs between the foci which are separated by tens of microns, not only photodamage is alleviated but also confinement of larger particles up to 100 μm (known as macro-traps) has become possible^36,37,41^.

However, since the trap is formed between the two foci and slightly away from the beam focus (unlike the OT) the gradient forces and hence the stiffness values of most of these CP beam trapping methods are quite small^31,36,40^. Consequently, this could lead to some lateral movement and slow rotation of the object, especially in the case of macro-traps^41^. Moreover, their trapping stiffness is highly sensitive to perfect alignment, which is due to the *symmetry* requirements of the CP beam traps. As a result, not only the alignment of the dual CP beam methods could be challenging, but also accurate particle positioning and force measurements will be limited to the CP trapping configurations. This problem is exacerbated if the foci distance is increased to extend the particle manipulation range. This is due to the fact that for CP traps, the optical forces and hence trap stability is strongly dependent on the foci separation^31,36,43^. The questions that arise here are the following: can we modify the dual CP beam configuration to simplify the alignment complexity and consequently reduce trapping stiffness sensitivity to perfect symmetry? And is there a way to increase the trapping size limit in order to stably confine particles larger than 100 μm?

In a previous study, we addressed the first question by utilizing dual *Asymmetrical Counter-Propagating* (ACP) beams along with the use of low-NA components to form the optical trap^44,45^. We showed that the asymmetry introduced in the two CP beam system not only increases trapping stiffness (with respect to traditional CP beam traps) but also allows for the axial scattering forces to be balanced over a wide spatial length. This in turn granted stable trapping and particle manipulation over a millimeter-range where the trapping stiffness remained almost independent of foci separation.

In this paper we plan to address the second question regarding trapping larger particles, but to do so first we will discuss a significantly less complex setup to create ACP traps than presented before^44^. We will demonstrate why this system is substantially easier to align and shows much less sensitivity to misalignments. Next, the trapping properties of the proposed system are studied, showing its capability for easy long-range particle manipulation inside liquid media. Our findings indicate enhanced trap stiffness values compared to conventional symmetric dual CP traps, when similar experimental conditions are used. Later, we observe the applications of the proposed ACP beams system in trapping significantly larger objects, especially elongated biological samples such as *C. elegans* worms with different lengths (up to 450 µm), using optical forces only. Finally, we demonstrate how spectroscopic imaging techniques such as light-sheet fluorescence microscopy can be easily integrated in this stable trapping system to generate more detailed images, without specimen movement.

## Experimental setup and methods

In the ACP dual beam trapping system^44^, unlike the traditional symmetric CP beams setup, we utilize two different optics with very different focal lengths to create the trap. To this end, a lens with a long focal length of 15 cm (hence long Rayleigh range) is placed on one side of the sample, while a low-NA objective (0.25 to 0.4 depending on particle size) is placed on the other side. So, while one side creates a relatively tight focus (OT like) the other side generates an almost collimated beam within the sample chamber, propagating in the opposite direction. In such a system, while the objective allows for 2D confinement (laterally), the two ACP beams work together to balance scattering forces (axially), creating a stable 3D trap. Since one of the two beams acts collimated within the sample, as its counter propagating beams’ tighter focus translates, the trap shifts without noticeable stability change for hundreds of microns, as shown later. Note that due to the low-NA objective used, thermal effects are avoided.

**Figure 1:**
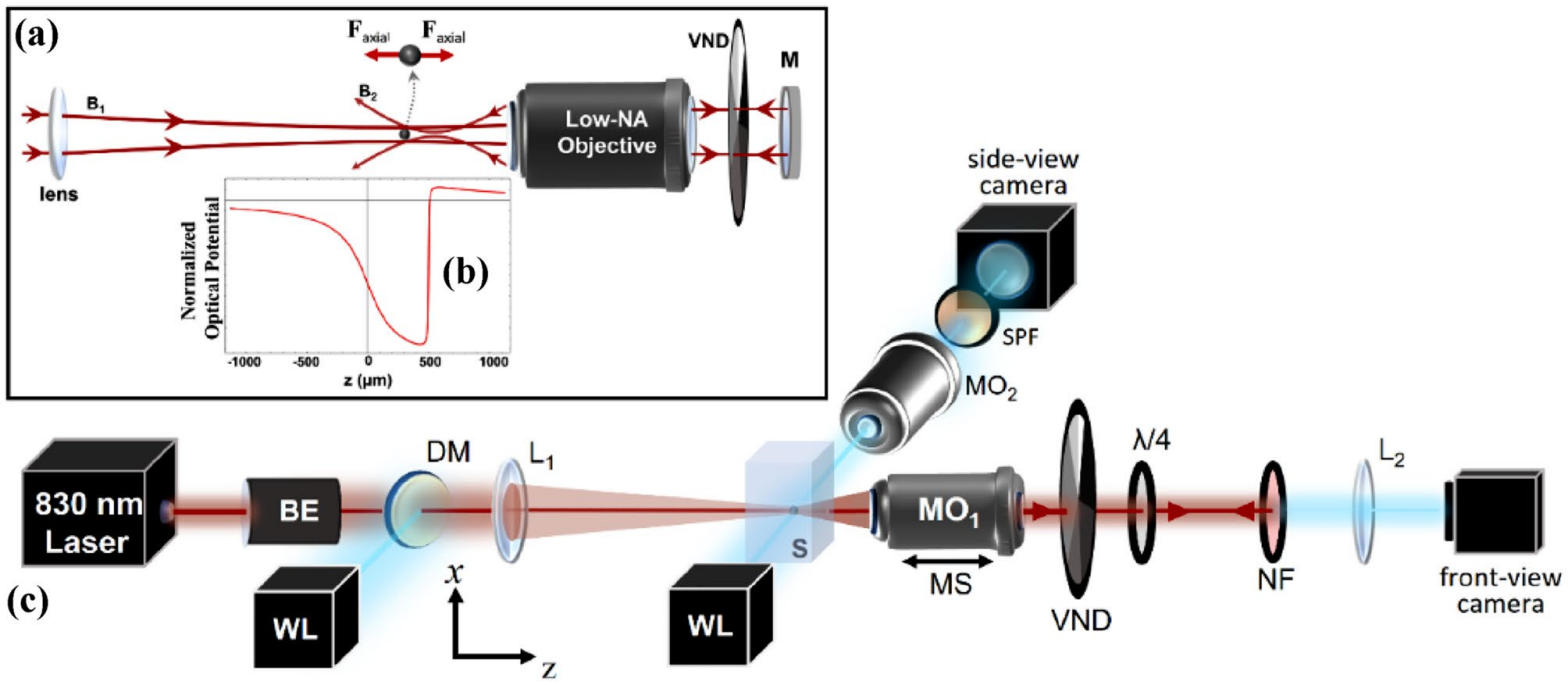
Retro-reflecting ACP trap setup with one incoming beam: (**a**) the basic idea of ACP trap formation: the only incoming beam entering the setup from the left, passes through the lens to create a loose focus with a long Rayleigh range which is collimated by a low-NA microscope objective and folds back on itself by the end mirror (M). VND is Variable Neutral Density filter wheel. (**b**) Is a simulation of the ACP system, demonstrating the formation of a potential well for the asymmetric trap, when the objective focus is 500 µm away from lens focus. (**c**) The retro-reflecting ACP setup used in our experiments: 830 nm Laser (MSquared Equinox SolsTiS PI), L_1_ and L_2_: two lenses with f_1,2_ = 15 cm, L_1_ in combination with MO_1_ (Olympus 20×/0.40 or Olympus 10×/0.25 depending on particle size) creates a 3D trap based on a single retro-reflecting ACP beam. If needed MO_1_ allows for frontal view and tracking, where L_2_ is used for frontal imaging. MO_2_: 4× (Olympus 4×/0.1) to 20× (Olympus 20×/0.40) objective used for side-view. MO_1_ is mounted on a motorized stage (MS) which moves axially. *BE* beam expander, *WL* white light, *NF 830 nm* Notch Filter which reflects 830 nm beam, *DM* dichroic mirror, S: sample and a quarter wave plate ($\lambda /4$) to change the beams polarization by 90° after passing through it twice. The two cameras are CMOS cameras.

In this work, to generate the ACP trap, we use a more simplified system compared to our previous work^44^ that uses only one incoming laser beam, as observed in Fig. 1a, described in the following. A laser beam, entering the system from the left is focused by a lens (fl = 15 cm), forming the first focus with a long Rayleigh range (z_r_ ≈ 650 µm). This beam is then collected by a low-NA microscope objective and directed to the end mirror (M) and folds back on itself, while passing twice through a variable neutral density filter wheel (VND) on its way, allowing power adjustments. The retro-reflected beam goes straight into the back aperture of the same microscope objective and comes to a tighter focus (the second focus) somewhere within z_r_ of the first focus, while propagating in the opposite direction forming the ACP beam trap. Each beam alone is unable to trap a particle, rather pushes it away. Only through the synergy of the two beams, we can generate a tight 3D trap which can occur at any point within and beyond z_r_, controlled by the objective location. Figure 1b shows the potential landscape generated by the ACP beams. To obtain Fig. 1b we computed the radiation pressure due to all incident beams as ${\mathrm{S}}_{\mathrm{z}}/\mathrm{c}$, where ${\mathrm{S}}_{\mathrm{z}}$ is the total Poynting vector in the axial direction and $\mathrm{c}$ is the speed of light. To obtain the potential landscape of Fig. 1b, we performed a path integral of the radiation pressure in the $\mathrm{z}$ direction from an arbitrary reference point to the location of interest. The result is a potential whose gradient yields the radiation pressure at each point on the optical axis. Figure 1c demonstrates the setup used in our lab to create the ACP trap which includes both front- and side-view imaging. Here, in order to have front-view imaging, we replace the mirror in Fig. 1a with a notch filter (NF) which acts like a mirror for the laser beam forming the trap, while transmitting all other wavelengths. The objective (MO_1_) is responsible for both front-view imaging and generating the tighter focus for optical trapping. For this reason, it sits on a motorized stage, so the exact location of its focus is in our control. Also, a quarter-waveplate ($\lambda /4$) is placed in the system such that it changes the polarization of the retro-reflected beam, preventing the formation of standing waves. Compared to the conventional dual CP traps, our setup is significantly less complex, easier to align and more robust to slight misalignments with an overall larger axial trapping stiffness as shown later. These are mainly due to the use of a single retro-reflected beam, low-NA optics and the broken symmetry of the beams forming the trap, about its centre.

There are many studies using a retro-reflected CP beam to trap and manipulate either nanoparticles^21,24,35–39^ by generation of standing waves or micro-particles^36,40,41,43^ between two tight foci tens of microns apart. However, they demonstrate relatively low values for the trap stiffness that is highly dependent on foci separation^31,36,43^. This in turn limits the manipulation range to tens of microns at most and the low stiffness values results in particle movement and rotation which can reduce image quality in certain microscopy techniques which require longer scanning times. As we will see shortly, the proposed ACP trapping system here, not only increases the trap stiffness, but also keeps its value almost independent of the foci distance, consequently allowing for the trapping, delivery or imaging of larger particles and microorganisms without any movement.

## Results, discussions, and further applications

**Figure 2:**
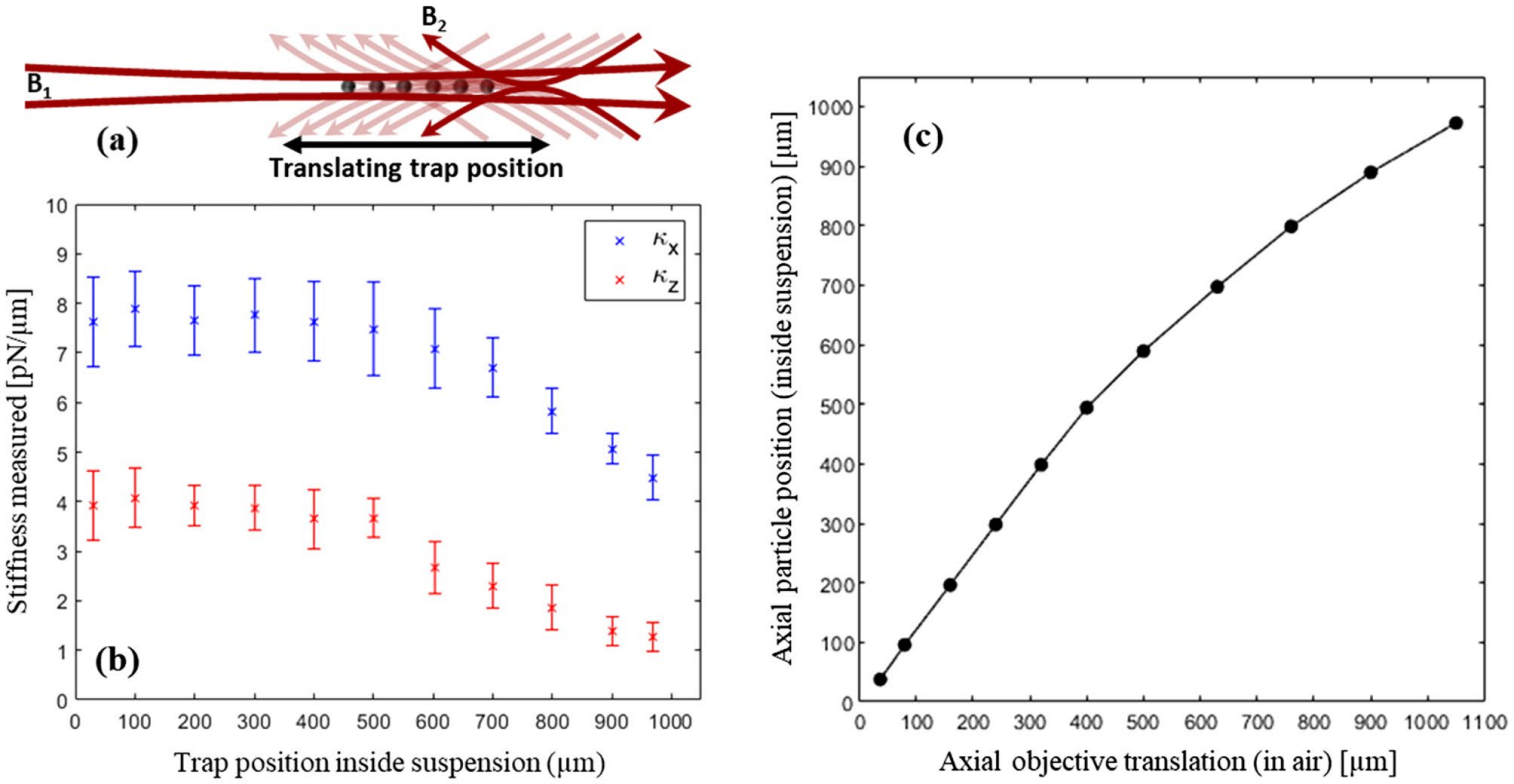
Particle translation behaviour: (**a**) A schematic demonstration of the trap position translation by moving objective MO_1_. (**b,c**) Experimental results of a trapped 5 µm particle (polystyrene bead) translation in water along the ACP beams as MO_1_ is translated by 100/1.33 μm steps in free space (which refers to 100 μm in water). (**b**) Axial and lateral trap stiffnesses measurements as a function of trap position inside the suspension for the confined 5 μm particle. (**c**) Experimental relationship found between axial objective translation in air and axial particle position inside the suspension.

For the conventional CP trapping systems in general, due to lower NA optics utilized (with respect to OT) the gradient forces are smaller, especially since the trap is formed in-between the foci due to perfect symmetry. These forces are even weaker if the foci separation is increased to tens of microns to increase manipulation range^31,36,43^. In this case, axial trapping is merely due to the balancing of the scattering forces. However, in the case of ACP traps, due to the broken symmetry of the beams, the stiffness values can be significantly improved by judiciously tuning the power ratios such that the trap is formed not somewhere between the foci, but rather at the location of the tighter focus. In this case while axial scattering forces are still balanced, the lateral gradient forces felt by the particle are much stronger because of its vicinity to the tighter focus. This is due to one beam being almost collimated (for hundreds of microns) while the other is focused much tighter, diverging quickly, thereby reducing its radiation pressure away from its focus. Consequently, by adjusting the power ratios we can create the axial balance at the location of the tighter focus, and as this focus is translated (by moving the objective) so is the trap’s position, as schematically illustrated in Fig. 2a. Here, the axial location of the potential well (trap position) generated by ACP beam is completely dominated by the position of the tighter focus as shown in Fig. 1b. In this figure, the lens focus is at position zero while the objective focus is 500 µm away and as illustrated in the plot the generated potential well location coincides with the objective’s focus.

Utilizing the setup shown in Fig. 1c where L_1_ has fl = 15 cm and MO_1_ is a 20× objective with NA = 0.4, we generated the ACP trap to confine a 5 µm polystyrene bead inside a 1 mm wide quartz cuvette filled with water. The laser powers used are 50 mW and 25 mW out of lens (L_1_) and objective (MO_1_), respectively. Lower powers can be used but that reduces the trap stiffness. Once a particle fell into the trap (which coincided with the location of MO_1_’s focus) it could be translated forward and back along the ACP beams by moving MO_1_. Experimental measurements of a trapped particle’s displacement behavior due to the objective’s axial translation, and the trap’s stiffness value at different locations in a 1 mm length are demonstrated in Fig. 2b,c. In plot (b), the lens focus was fixed at the location of the left-side wall of the cuvette and the objective was moved with respect to this wall in 100 µm/n_water_ increments outside of the sample chamber and towards the right, where n_water_ = 1.33. The increment value corresponds to a 100 µm displacement of MO_1_’s focus inside water. As shown in Fig. 2c the trapped particle follows MO_1_’s displacement with an almost one-to-one ratio up to position 500 µm inside the sample. After that it starts deviating and the particle translates slightly less, and the line starts bending. This deviation happens because as the particle is moved further away from the focus of the lens, the scattering forces (axial radiation pressure) imposed on it by the moderately focused beam (by lens L_1_), starts to drop more noticeably. Consequently, this will lead to the trap forming slightly away from the tighter focus which is where the axial forces balance.

We also characterized the ACP trap stability as a function of the particle location inside the sample chamber, by measuring the axial and lateral stiffnesses (κ_z_, κ_x_). Figure 2b illustrates the results of trap stiffness measurements after every 100 µm of particle axial translation inside the suspension, for up to 1 mm length. In these measurements, the PSD (power spectrum density) method was used, and at each location the measurements are repeated 10 times for both axial and lateral directions and averaged in order to find κ_z_ and κ_x_, respectively. The PSD is found by tracking the particle for 10 s, using a side-view CMOS camera with 1000 fps. The first and last data points are measured 25 µm away from the wall to avoid surface effects. As observed in Fig. 2b both axial and lateral stiffness values remain almost constant as we move the trap position up to ~ 500 µm. In this range the average measured stiffness values are κ_x_ = 7.7 pN/µm and κ_z_ = 3.8 pN/µm which are about one order of magnitude larger than the conventional CP trap stiffnesses reported previously^6,36,40,41^ where similar experimental parameters have been used. The reason for this boost in stiffness values we demonstrate here is due to the asymmetry of our system which causes the trap to form very close to the objective’s focus, resulting in enhanced gradient forces felt by the particle which is independent of foci separation of up to ~ 500 µm. For the conventional CP traps, due to perfect symmetry, the trap is formed in the middle of the foci and depending on their distance, the gradient forces on the particle and consequently stiffness values could be significantly smaller. As we translated the particle further out of the lens’s Rayleigh range, it remained confined, but the measured trap stiffnesses dropped. This result is in agreement with the line bending observed in plot 2c and is due to the trap location deviating away from the objective’s focus as the particle is translated further away from the lens’s Rayleigh range. This deviation reduces the gradient trapping forces felt by the particle, leading to a decrease in stiffness values.

Using the exact same setup, we were able to trap and translate polystyrene particles with diameters between 1 and 100 µm, inside aqueous suspension. This capability of the system along with the results presented in Fig. 2 demonstrates the flexibility of the ACP system in particle trapping and manipulation. The ability to translate and control the trap’s axial location with micron precision for several hundreds of microns is quite unique. These properties are very different from the conventional CP traps where one can only move a trapped particle for tens of microns before it escapes the trap. Also, in the case of symmetric CP traps, if the focus of one objective moves by distance d inside the sample, the trapped particle moves by d/2. All these differences arise from the asymmetry introduced here.

### Retro-reflected ACP traps vs the dual CP

In order to further investigate the properties of the retro-reflected ACP traps and how they differ from the conventional CP traps, we theoretically modelled both systems’ axial forces using parameters similar to those used in our experiments. The results of this study are briefly discussed here and can be found in more details in Supplementary Fig. S1. Our calculations show that for the ACP trap, even at large foci separations (of more than 500 µm), the particle can still be confined due to the existing strong optical forces. This result is in agreement with our experimental results presented in Fig. 2 and explains why we are able to easily translate a trapped particle for hundreds of microns. On the other hand, our theoretical findings for the symmetric CP trap are in contrast with those of ACP trap (with comparable lateral stiffness values). For the symmetric CP trap, if the foci separation becomes larger than tens of microns, their axial forces become too small, and a stable 3D trap becomes impossible. This result is in agreement with previous reports regarding conventional CP traps where their trapping stiffness is a strong function of foci separation^36,43^ and drops rapidly as foci separation increases. This in turn limits the manipulation area to tens of microns for CP traps. In another theoretical study, we investigated both ACP and CP trap translation capability by only changing the power ratios of the two sides. Our results shown in Supplementary Fig. S1a indicate that changing the power ratios in either case will move the trap location by tens of microns only. Overall, our findings from the axial force study suggest how the asymmetry introduced to the conventional CP trapping system can significantly extend the optical force range, and consequently increase our control over the exact trapping location within a millimetre path.

### Trapping larger round and elongated micro-organisms with ACP traps

**Figure 3:**
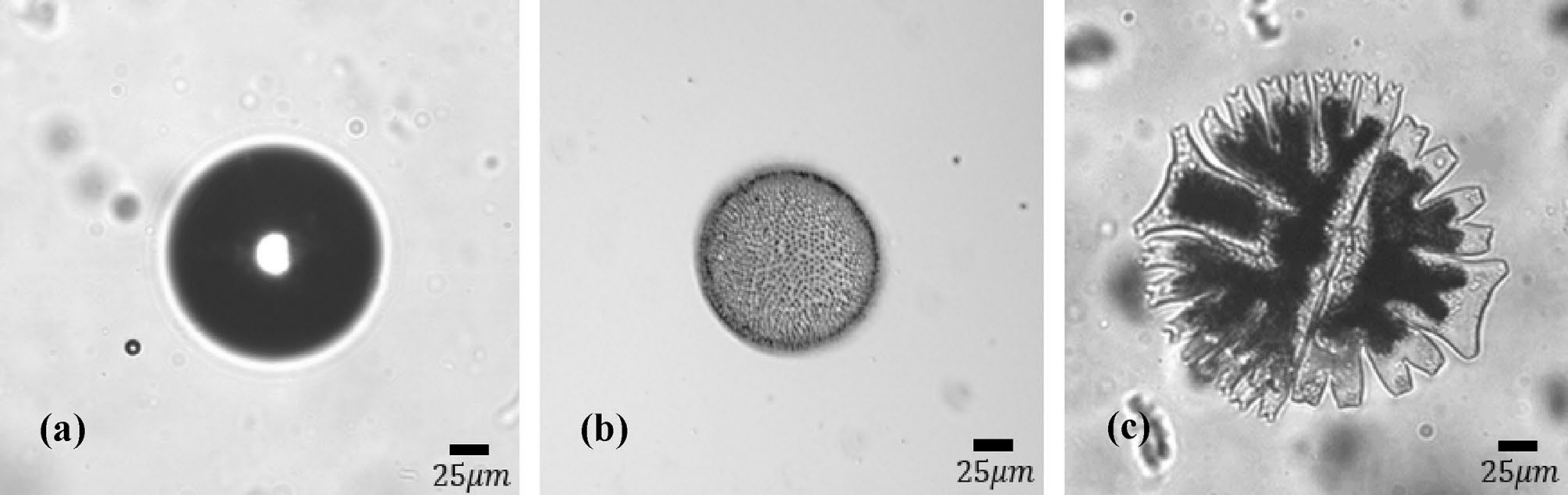
Trapping of much larger micron-size objects using the retro-reflected ACP trapping system. Confinement of: (**a**) 150 µm polystyrene bead, (**b**) 115 µm Volvox and (**c**) 250 µm Micrasterias Waris living microorganisms.

So far, we have only demonstrated the confinement of small particles using the retro-reflected ACP trap proposed. However, as we will soon observe, this technique is very suitable for trapping significantly larger macro-sized particles which can be spherical or non-spherical. In this section, we will start with 3D trapping of large polystyrene beads then we will demonstrate confinement of living biological samples. First using the setup in Fig. 1c (where MO_1,2_ are 10× objectives with NA of 0.25) we were able to trap 150 µm polystyrene beads inside an aqueous suspension as illustrated in Fig. 3a. The video of this confinement is in Supplementary Visualization 1. We added 10 µm polystyrene beads to the suspension, for comparison purposes as seen in Fig. 3a. We were also able to translate the 150 µm particle by slowly moving MO_1_. Sequence of images showing the translation of this bead is presented in Supplementary Visualization 2. The laser power used for trapping such large polymer bead was 100 mW in total: 67 mW from the beam exiting lens L_1_ and 33 mW from the retro-reflected beam exiting MO_1_ and entering the sample. It is important to note that in a previous research^48^ a 100 µm polymer sphere was trapped between two fibers with a trapping power of 800 mW from each fiber arm. Compared to that study, the power levels used in our study are significantly smaller and the particle trapped here is 1.5 times larger. The rather small power required for trapping, along with the capability of confining much larger objects indicates that the ACP beam trap is an ideal tool for trapping biological specimen.

Next, using the same setup and parameters, we were able to readily trap 115 µm Volvox (Fig. 3b) and 250 µm Micrasterias Waris (Fig. 3c) living microorganisms inside water. The video of trapped Micrasterias Waris is available in Supplementary Visualization 3.

**Figure 4:**
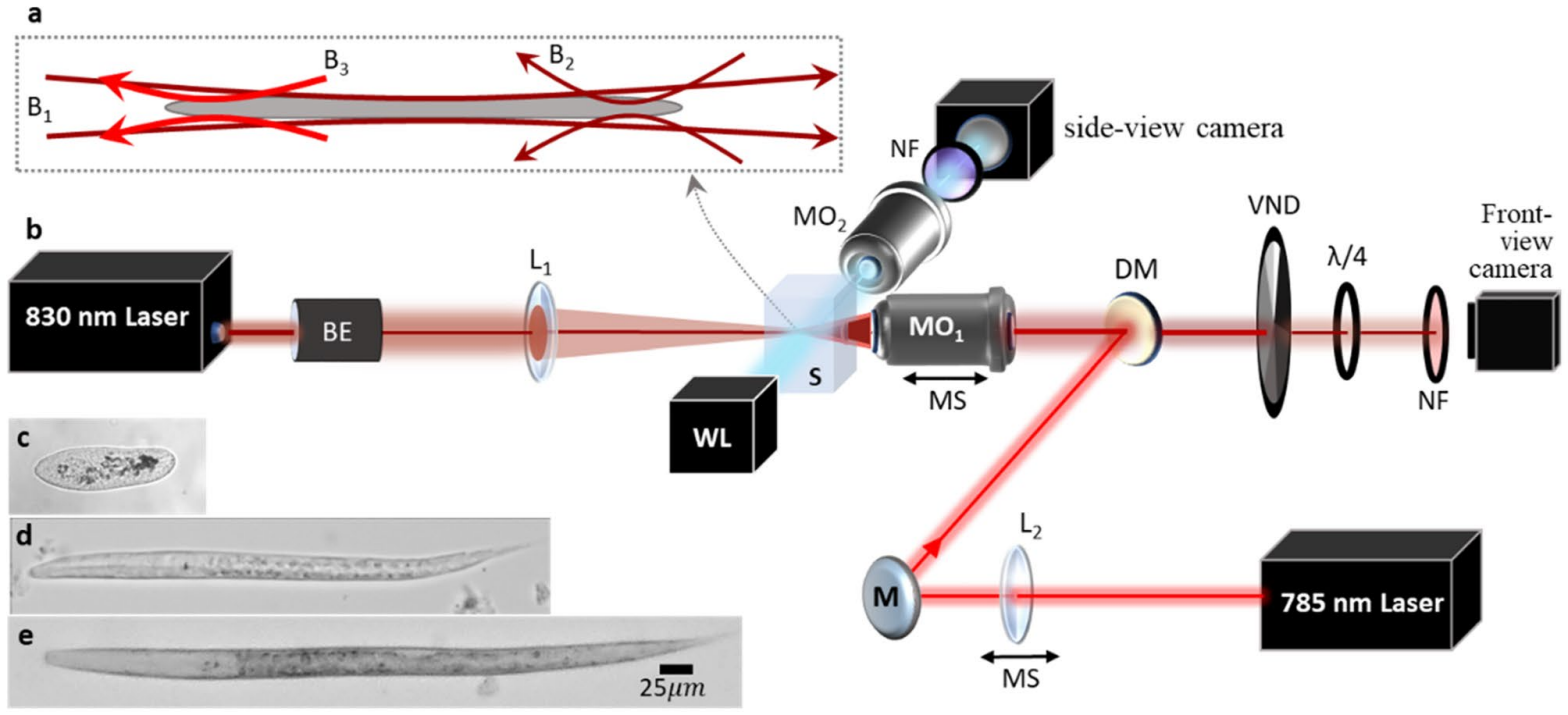
(**a**) The schematic of the proposed dual trap ACP beams to confine elongated objects using a combination of three beams. B_1_ is the 830 nm beam focused via L_1_ (fl = 15 cm) with a long Rayleigh range, B_2_ is the retro-reflected beam that is focused tightly via MO_1_ (Olympus 10×/0.25). B_3_ is a 785 nm laser beam also focused by MO_1_ slightly after the focus of B_2_. (**b**) Modified folding ACP beam setup to achieve 3D confinement for elongated objects. Here MO_1_ is 10× with NA 0.25, MO_2_ varied between 4×, 10× and 20× objective depending on object size and the application. (**c–e**) are the bright field images of optically trapped microorganisms: (**c**) 130 µm Paramecium aurelia, (**d**) 260 µm, (**e**) 385 µm *C. elegans* larvae.

Many biological samples are either elongated or rod shaped, such as chromosomes, intracellular organelles, a wide variety of bacteria and parasites, membrane tubules, certain microalgae and micro-worms. Optical trapping such samples usually requires complex systems involving beam shaping. Consequently, 3D optical trapping of rod-shaped particles in a specific orientation can be challenging or not possible. Here, by integrating a second laser beam into the retro-reflecting ACP trapping system, as shown in Fig. 4, we can easily trap elongated or rod-shaped objects as described in the following. The second beam, which is chosen at a slightly different wavelength (λ = 785 nm with ~ 30 mW) is combined with the retro-reflected 830 nm beam using a DM (dichroic mirror), both entering MO_1_ while copropagating. The two beams pass through the sample (B_2_ and B_3_ in Fig. 4a) focusing at two separate locations, where their distance can be simply controlled by moving lens L_3_ (fl = 15 cm which changes the divergence of the 785 nm laser beam). The combination of the three beams (B_1_, B_2_ and B_3_ with ~ 80 mW, ~ 30 mW and ~ 30 mW, respectively) forms a 3D trap for elongated objects. Using this system, we successfully trapped *Paramecium aurelia* (Fig. 4c) and *Caenorhabditis elegans* (*C. elegans*) micro-organisms (Fig. 4d,e). The *C. elegans* worms confined were in different larval stages with various sizes ranging between 250 and 450 µm in length. The media of a larva (stage L_2_) confined in our dual ACP trap, can be found in the Supplementary Visualization 4.

### Integration of light-sheet microscopy

**Figure 5:**
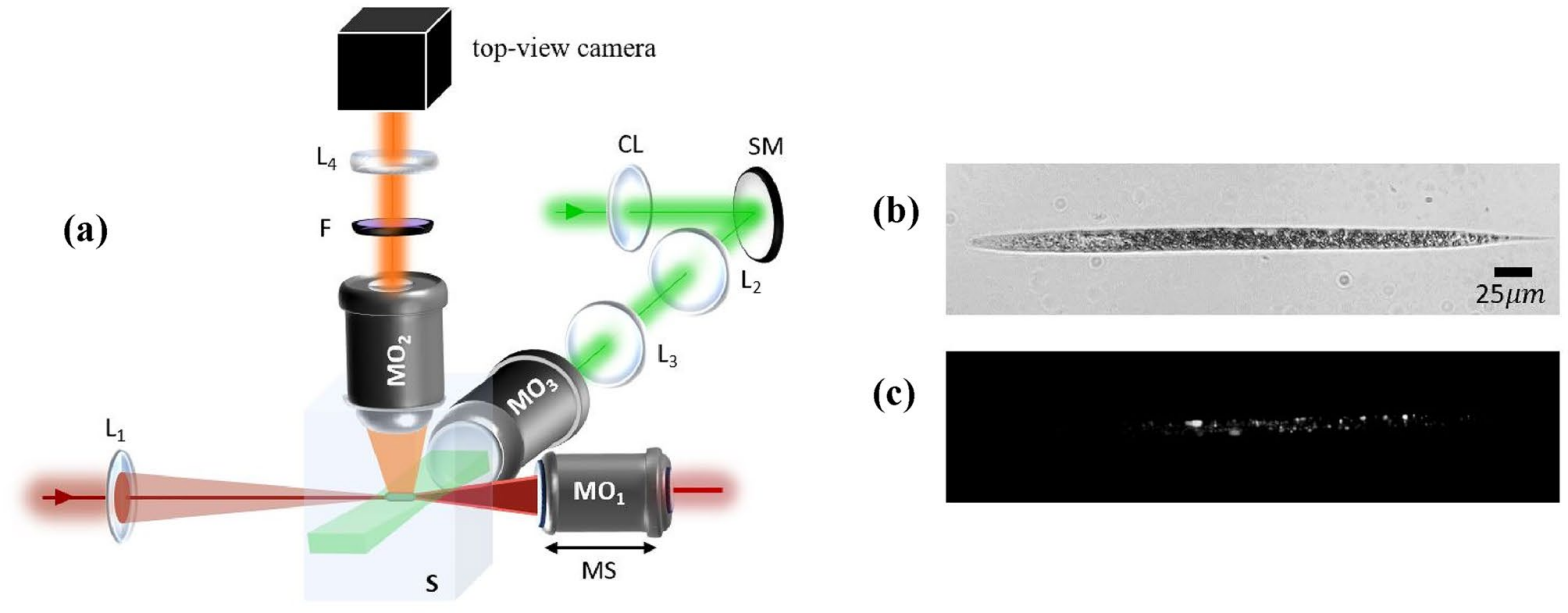
(**a**) The zoomed in optical setup showing only the objectives and lenses that are involved in optical trapping (L_1_ and MO_1_) and light-sheet fluorescence microscopy (MO_2_, MO_3_ and L_2_ to L_4_). Here: *CL* cylindrical lens with fl = 7.5 cm, MO_3_ is a 10× objective with NA of 0.3 (Olympus UPlanFL N 10×/0.30) while MO_2_ is 20× water immersion objective with NA of 0.6 (Thorlabs TL20X-MPL 20×/0.60 W/400–900 nm $\infty$/WD 5.5 mm). L_2_ and L_3_ are relay lens system both with fl = 5 cm. L_4_ with fl = 10 cm is used for imaging. (**b**) Is the bright field microscopy of a 450 µm stained *C. elegans* larva and (**c**) is the light-sheet fluorescence microscopy image of the same larva shown in (**b**).

Next, we added light-sheet fluorescence microscopy to the ACP trapping system illustrated in Fig. 4b for simultaneous 3D trapping and imaging of the trapped *C. elegans* larvae. For this microscopy, the lipid droplets in the larva were stained with the fluorescent dye Nile red (a lipophilic stain that fluoresces in a lipid environment). As demonstrated in Fig. 5a, we use a 532 nm laser to excite the confined stained *C. elegans* worm. The beam is focused by a cylindrical lens (CL with fl = 7.5 cm) onto a steering mirror (SM) and then relayed to the back aperture of MO_3_ objective (10× with NA of 0.3) by a relay lens combination (two identical lenses L_2_ and L_3_ with fl = 5 cm). The fluorescence is collected perpendicular to the illumination plane with a 20× water immersion microscope objective (MO_2_) with NA of 0.6 and imaged on the top-view CMOS camera. The bright field image of a 450 µm stained *C. elegans* larva is shown in Fig. 5b. An example of a sectional light-sheet image taken parallel to the plane the larva lies on, is presented in Fig. 5c.

It should be noted here that for smaller trapped objects (≤ 100 µm), we did not need to add MO_3_ for light-sheet microscopy, since MO_1_ could be used for both trapping and light-sheet formation. This could be easily achieved by utilizing a dichroic mirror to combine the excitation laser (here 532 nm) with the retro-reflected beam, before entering MO_1_. Here we have only demonstrated one possible imaging technique (light-sheet microscopy) which was easily integrated in our optical trapping setup. However, since our system, unlike most previous studies, allows objects of interest to be confined inside a much larger chamber and without a tight physical constraint (such as using a coverslip or agarose), it enables the integration of a variety of imaging techniques (with a large field of view) to better study bio-samples and their development. Consequently, our system has the potential for 4D imaging (3D imaging over time) in order to study specific dynamics in bio-matter.

## Conclusions

In summary, we have demonstrated how breaking the symmetry of the well-known counter-propagating optical trapping system can modify the overall optical forces leading to increased trapping stability and allowing for long-range particle trapping and manipulation in liquid media. While there are numerous reports on long-range particle manipulation via optical tweezers, most of them are performed in gas or vacuum media and mainly rely on thermophoretic forces^29,46,47^. Here we trap and manipulate a broad range of particles with different sizes and shapes, including microorganisms, with the use of radiation pressure forces and without creating standing waves^22^ or thermal effects. Due to the asymmetry around the trap created by the lens-objective combination, the sensitivity of the trap stiffness on foci separation is extremely reduced which in turn, allows for trapping at extended foci separation which is not possible with conventional CP traps. Moreover, the broken symmetry combined with the retro-reflection of the beam and the use of only one low-NA objective, have not only significantly simplified the alignment but also made it very robust and cost efficient. The proposed ACP setup has increased axial trapping stiffnesses by at least an order of magnitude with respect to the symmetric CP beams that have similar experimental parameters. While our setup shares the simplicity and flexibility of a single-beam optical tweezer, it allows for the use of objectives with very small NAs for particle trapping. This results in a larger working distance and field of view while avoiding the undesired thermal effects. This is of particular interest when far-field, non-invasive trapping and manipulation is desired inside liquid media. All these advantages make this system very practical for long-range optical trapping for a variety of samples and allow for the possibility of integration of spectroscopy-based microscopy techniques, as demonstrated here. It is worth mentioning that in the past few years, techniques such as optical mirror traps^36,37^ (or optical macro-tweezers^41^) all use a mirror behind the sample to form counter propagating trapping beams but there are major differences. While most of these studies use a spatial light modulator (SLM), making their setup quite complex and expensive, the retro-reflecting mirror used is placed within the sample chamber making both sample preparation and side-view imaging quite challenging. In our proposed method the mirror (which is a NF) is placed far from the sample allowing for side-view access without the need for special sample preparation or complicated imaging techniques. Additionally, the unique asymmetry of our trapping beams creates stable millimetre-range 3D trapping and manipulation capabilities with enhanced stiffness values absent in the aforementioned studies due to their beam symmetry.

## Supplementary Information

Supplementary Information 1.

Supplementary Legends.

Supplementary Information 2.

Supplementary Information 3.

Supplementary Information 4.

Supplementary Information 5.

## Data Availability

Data are not publicly available but can be obtained from Shima Fardad upon reasonable request.
