# Supplementary material for: Optical trapping of sub-millimeter sized particles and microorganisms
Source: Sci Rep. 2023 May 27;13:8615. doi: 10.1038/s41598-023-35829-7 (PMC10224970; doi:10.1038/s41598-023-35829-7)
Supplement: Supplementary file 1 — Supplementary Information 1. [file 41598_2023_35829_MOESM1_ESM.docx]

Optical Trapping of Sub-millimeter Sized Particles and Microorganisms

# Laurynas Lialys^1^, Justinas Lialys^1^, Alessandro Salandrino ^1,2^, Brian D. Ackley ^3^, and Shima Fardad^1,2,*^

^1^Department of Electrical Engineering & Computer Science, University of Kansas, Lawrence, 66045, USA

^2^I2S, Institute for Information Sciences, University of Kansas, Lawrence, 66045, USA

^3^Department of Molecular Biosciences, The University of Kansas, Lawrence, 66045, USA

^*^sfardad@ku.edu

In order to further investigate the properties of the retro-reflected ACP traps and how they differ from the conventional CP traps, we theoretically compared their axial forces for different foci separation. Fig. S1. illustrates the results for these calculations, where the normalized axial forces in Fig.S1 are obtained by evaluating the radiation pressure$S_{z}/c$, where $S_{z}$ is the axial component of the Poynting vector due to the superposition of the incident beams. We have considered z_r, lens_=650μm and z_r obj_=3μm in free space, z_r_ represents the Rayleigh length of the lens and objective forming the trap. The foci separations used here are 500μm and zero in (a) and (b), respectively. We have considered a power ratio of 2 between the ACP beams, where the beam entering the lens has twice the power of the beam entering the back aperture of the objective which is the same ratio used in our experiments. In this approximation we assume that the scattering forces are directly proportional to the intensity on the beam axis, and we ignore interference.


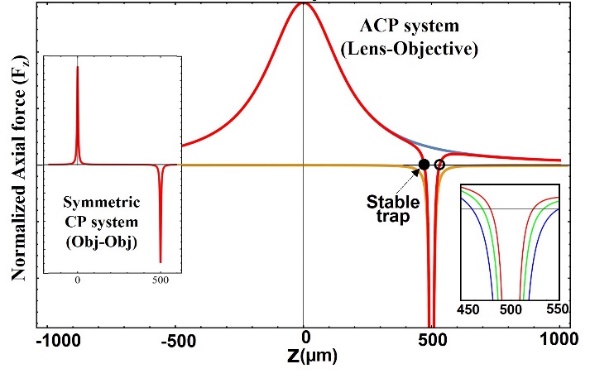

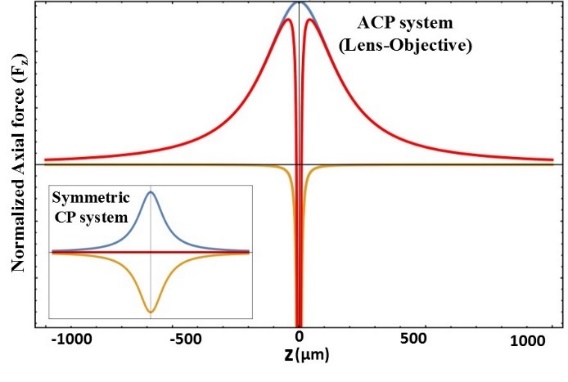


**(a)**

**(b)**

Fig. S1. Comparison of axial forces in symmetric and asymmetric CP traps: (a) The red curve shows the sum of axial optical forces due to the ACP beams on a particle anywhere between the two foci which are 500μm away from each other. The z_r lens_=650μm and z_r obj_=3μm forming a stable trap. Insert graph on the left shows axial forces of CP beams generated by two identical objectives (NA=0.4, z_r obj_=3μm) when the foci are 500μm apart. Right insert demonstrates how increasing the power on the objective side by 2X and 4X with respect to its original value (red plot) can shift the trap location by only tens of microns, shown in green and blue plots, respectively. (b) Is the same calculation as (a) but the foci are overlapped. In both plots, the blue and yellow curves represent axial forces of the focused beam from the lens and objective, respectively.

Fig. S1.a. shows that there are two locations where trapping can occur for this ACP trap. Those are where the sum of axial forces cancels out, marked with full and hollow circles. Nevertheless, only the full circle location forms a stable trapping position, for the following reason. If a particle is trapped at this location and moves slightly to the left or right (due to Brownian motion), the axial optical forces of the system will push it right or left, respectively and back into the trap. However, for the hollow circle position, if the particle slightly moves, the optical forces will completely push it away from the trap. Another result from Fig. S1.a. is that even at large foci separations for ACP beams, the particle can still be trapped as we mentioned before. This result is in contrast with the conventional CP beams where trapping stiffness is a strong function of foci separation [1,2]. For symmetric CP beams with comparable lateral stiffnesses, if the foci separation becomes this large, then their overlap vanishes such that axial forces become too small at the centre as seen in the left insert of Fig. S1.a. This makes 3D trapping impossible.

The trap translation by changing the power ratios of the ACP beams (via VND) is also calculated. As seen in Fig. S1.a. (right insert) by increasing the power on the objective side by a factor of 2 and 4 (shown in green and blue curves correspondingly) with respect to its original value (red curve) the trap shifts by only tens of microns. While moving the objective forward and back, one can move the location of the 3D trap along the beams by hundreds of microns (up to millimetre range), changing the power ratios will only allow fine tuning the trap location. For the symmetric CP beams, trapping is only achievable when the foci are very close, and the trap position cannot be translated more than tens of microns [1,3,4,5]. On the other hand, if the foci separation of the symmetric CP beams becomes zero (i.e., the foci overlap) then the scattering forces in the axial direction cancel out entirely along the beams, resulting in no trapping forces (red line in the insert of Fig. S1.b.). This is contrary to the ACP system calculation shown in Fig. S1.b., where overlapping foci demonstrate a nonzero force and 3D trapping is possible, which has also been experimentally observed. The graphs in Fig. S1. illustrate how the asymmetry introduced to the conventional CP trapping system can significantly extend the optical force range, and consequently increase our control over the exact trapping location within a millimetre path. As we will demonstrate shortly, ACP beams not only allow for extended 3D trapping but also their trap stiffness values are at least one order of magnitude larger than the conventional CP traps.

The normalized axial forces in Fig.S1 are obtained by evaluating the radiation pressure$S_{z}/c$, where $S_{z}$ is the axial component of the Poynting vector due to the superposition of the incident beams.

# References

1. Pitzek, M., Steiger, R., Thalhammer, G., Bernet, S. & Ritsch-Marte, M. Optical mirror trap with a large field of view. *Opt Express* **17**, 19414-19423 (2009).
2. Rodrigo, P. J., Perch-Nielsen, I. R. & Gluckstad, J. Three-dimensional forces in GPC-based counterpropagating-beam traps. *Opt Express* **14**, 5812-5822 (2006).
3. Ashkin, A. Acceleration and Trapping of Particles by Radiation Pressure. *Phys Rev Lett* **24**, 156-& (1970).
4. Lindballe, T. B. *et al.* Three-dimensional imaging and force characterization of multiple trapped particles in low NA counterpropagating optical traps. *J Eur Opt Soc-Rapid* **6** (2011).
5. Yang, Z. Y., Piksarv, P., Ferrier, D. E. K., Gunn-Moore, F. J. & Dholakia, K. Macro-optical trapping for sample confinement in light sheet microscopy. *Biomed Opt Express* **6**, 2778-2785 (2015).
6. Bowman, R. *et al.* Position clamping in a holographic counterpropagating optical trap. *Opt Express* **19**, 9908-9914 (2011).
