# Supplementary material for: Optical trapping of sub-millimeter sized particles and microorganisms
Source: Sci Rep. 2023 May 27;13:8615. doi: 10.1038/s41598-023-35829-7 (PMC10224970; doi:10.1038/s41598-023-35829-7)
Supplement: Supplementary file 2 — Supplementary Legends. [file 41598_2023_35829_MOESM2_ESM.docx]

**Supplementary legends**

Visualization 1: A trapped 150µm polystyrene bead inside an aqueous suspension using our Asymmetrical Counter**-**Propagating (ACP) trapping technique

Visualization 2: Sequence of images showing the translation of 150µm polystyrene bead inside an aqueous suspension using the ACP trapping system

Visualization 3: A trapped 250µm Micrasterias Waris inside an aqueous suspension using the ACP trapping system

Visualization 4: A trapped L2 stage *C. elegans* larva inside an aqueous suspension using the dual ACP trap

.
