# Supplementary figures and images for: Optical trapping of sub-millimeter sized particles and microorganisms
Source: Sci Rep. 2023 May 27;13:8615. doi: 10.1038/s41598-023-35829-7 (PMC10224970; doi:10.1038/s41598-023-35829-7)

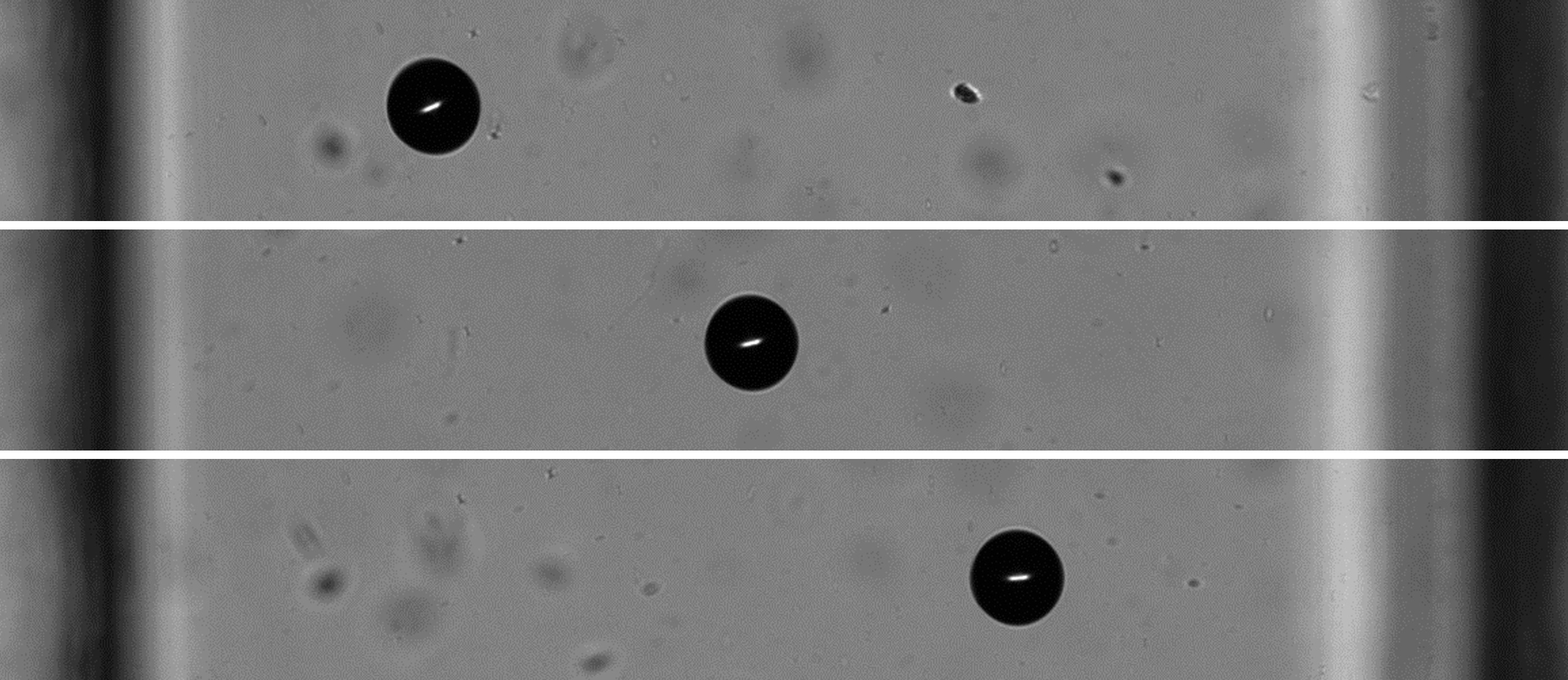

Supplement: Supplementary file 4 — Supplementary Information 3. [file 41598_2023_35829_MOESM4_ESM.jpg]
